# Juvenile Myoclonic Epilepsy Presenting with Neurocognitive Impairment: A Case Report

**DOI:** 10.7759/cureus.2271

**Published:** 2018-03-05

**Authors:** Sarfraz Mahesar, Hira F Akbar, Husnain Abid, Rabia Sana

**Affiliations:** 1 Department of Neurology, Dow University of Health Sciences (DUHS), Karachi, Pakistan; 2 Civil Hospital Karachi, Dow University of Health Sciences (DUHS), Karachi, Pakistan; 3 Student, Dow University of Health Sciences (DUHS), Karachi, Pakistan

**Keywords:** juvenile myoclonic epilepsy, cognitive dysfunction, neuropsychological assessment

## Abstract

Juvenile myoclonic epilepsy (JME) is a genetically and clinically diverse disorder which is characterized by myoclonic jerks, usually after awakening from sleep. It affects both genders equally and manifests during the second decade of life. The various precipitating factors include stress, light, sleep deprivation, and alcohol. A history of morning clumsiness supported by typical electroencephalography (EEG) findings, together with a normal clinical examination all point towards a diagnosis of JME.

We present the case of a nine-year-old girl who presented with cognitive dysfunction in addition to myoclonic jerks. She had normal brain imaging and her labs were negative for other causes of dementia. Her EEG findings revealed polyspikes with normal background activity. She was treated with antiepileptic drugs (AEDs) for control of seizures.

## Introduction

Approximately 70 million people are affected with epilepsy around the world. Though proper epidemiological studies do not exist for Pakistan, it is estimated that the prevalence of epilepsy in Pakistan is 9.99/1000 [1]. Juvenile myoclonic epilepsy (JME) is an idiopathic generalized epilepsy syndrome with onset in adolescence; it accounts for 5%-10% of all epilepsies [2]. The clinical hallmark of this syndrome is myoclonic jerks, with or without generalized tonic-clonic seizures and/or absence seizures. These seizures can be triggered by factors such as alcohol consumption, stress, fatigue, sleep deprivation, continuous flickering of lights, and menstruation [3]. Genetic factors play a significant role in the development of this syndrome and it is associated with a strong family history [4]. Definitive diagnosis is made on the basis of a typical patient history and electroencephalography (EEG) findings [3] with normal brain imaging. Even though patients respond well to the pharmacological treatment, there is a high rate of recurrence with the discontinuation of antiepileptic drugs (AEDs) [5].

This case report is about a nine-year-old girl with peculiar neurological symptoms. The patient was diagnosed with JME and was successfully treated with valproic acid. The purpose of this case report is to raise awareness about JME and its complex presentation, which may help clinicians to promptly diagnose such cases using EEG and to achieve an improved response with AEDs.

## Case presentation

History

A nine-year-old girl presented to the Neurology Department of the Civil Hospital, Karachi, Pakistan with complaints of forgetfulness and morning clumsiness for the past two months. The patient started losing interest in daily homework and playing with other classmates and became quite reluctant to attend school. Teachers noticed that the patient faced difficulty in learning and memorizing new lessons. She later totally isolated herself from friends and showed lack of interest in daily activities.

A week after the memory loss, she developed jerking of the right arm. Jerks were unilateral, repetitive, would last for a few seconds and usually occurred in the morning, two to three times per day. Her consciousness was intact with no up-rolling of eyeballs, frothing, or tongue biting. Her responses to simple commands like “open your mouth" or "close your eyes” were observed. There was no associated fever, photosensitivity, hearing loss, diplopia, limb weakness, paraesthesia, or urinary incontinence. She visited multiple general practitioners (GPs) and local faith healers, but the treatment failed to show any improvement. The developmental history was normal and she attained normal milestones. The patient had four siblings and there was no family history of diabetes mellitus or ischemic heart disease, but epilepsy was reported on the maternal side of the family. There was no history of tuberculosis or its close contact.

Examination

On general physical examination, the patient had an average height and build, was lying in bed comfortably, and was oriented with time, place, and person. Higher mental function and speech was normal. The mini mental state examination (MMSE) of the patient was 23 with no signs of meningeal irritation. Both pupils were equally reactive to light with normal extraocular muscle movement present in all directions of gaze. All other cranial nerves were intact. The motor examination was normal, with a Medical Research Council (MRC) score of 5 in all limbs. Sensory examination was also normal with a standard cerebellar and sensory system.

Investigation

**Figure 1:**
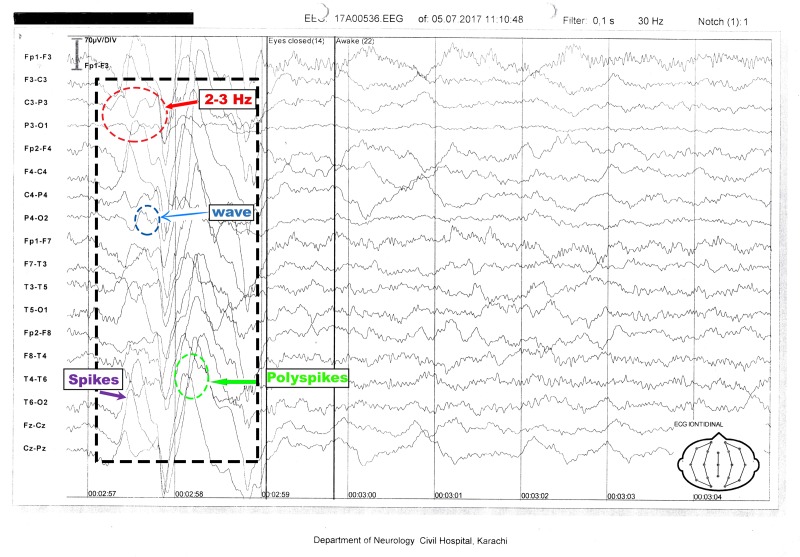
Electroencephalography tracing of a nine-year-old, awake patient Electroencephalography (EEG) shows generalized spike waves and polyspike of 2-3 Hz along with a normal alpha rhythm background.

The patient's baseline investigations included complete blood count (CBC), liver function tests (LFTs), serum electrolytes, folate and thyroid stimulating hormone (TSH) levels, Vitamin B12 levels, fluorescent treponemal antibody absorption (FTA-ABS), and treponema pallidum hemagglutination assay (TPHA), all of which were within normal ranges. Her viral markers and autoantibody profile were also negative. Magnetic resonance imaging (MRI) scans of the brain, cerebrospinal fluid (CSF) analysis, and abdomen ultrasounds were unremarkable. The EEG showed generalized discharges of spikes and polyspikes of frequency 2-3 Hz with a normal background of alpha wave rhythm (Figure 1 ).

Treatment

The patient was started on valproic acid; doses were gradually increased to therapeutic levels. She was counselled for proper sleep and proper dietary habits. After discharge, the patient was referred for rehabilitation and psychotherapy and was followed up on monthly basis. The patient showed a good response to the treatment and her MMSE score improved significantly to 28.

## Discussion

Koepp et al. [6] described the seizures in JME to be bilateral. But the patient experienced only right side seizures at an unusual age. Therefore, differential diagnoses of JME, central nervous system (CNS) infections like encephalitis, progressive myoclonic epilepsy (PME) and mitochondrial myopathy, encephalopathy, lactic acidosis, and stroke-like episodes syndrome (MELAS) were made.

Although the general clinical and EEG characteristics had been widely known since the 20th century [7], JME was very frequently misdiagnosed. Panayiotopoulos et al. [8] described the major reasons for misdiagnosis as unilateral jerks, nocturnal generalized tonic-clonic jerks, and focal EEG abnormalities. Therefore, it is important for primary care physicians to differentiate JME from other epileptic diseases like PME, as both present with similar clinical findings. In PME, progressive neurologic impairment and signs of cerebellar dysfunction like ataxia are found [9]. The EEG pattern is also different for the epileptic syndromes. While the MRI and computed tomography (CT) scans of the brain are almost always normal in JME, these imaging techniques usually show diffuse cortical atrophy without any apparent parenchymal alterations in PME [9]. A histopathological examination on biopsies of various tissues like brain, skin, muscle, and liver is required for a definitive diagnosis of PME [9]. A diagnosis of JME is usually made on the basis of EEG findings and its results can range from a completely normal EEG to lateralized or focal discharges [10]. The typical EEG pattern shows 3-5 Hz generalized discharges of spike waves and polyspikes with normal background activity. However, the frequency of complexes can be as slow as 2 Hz to as fast as 7 Hz. Park et al. stated video-EEG monitoring (VEM) as a valuable tool in the diagnosis of JME with a diagnostic accuracy of 50% [10].

The gold standard choice of treatment for JME is lifelong antiepileptic drugs (AEDs), such as sodium valproate. A limited number of studies have also reported levetiracetam, lamotrigine, topiramate, and zonisamide to achieve seizure control [6]. Carbamazepine and oxcarbazepine are contraindicated as they may increase the risk of myoclonic seizures and might precipitate generalized tonic-clonic seizures. Recently, a non-enzyme-inducing sodium channel blocker, lacosamide has been used as adjuvant treatment for the control of seizures. It has also been used as an add-on therapy in patients suffering from PME type 1, leading to a significant decrease in tonic-clonic seizures and improved cognition [5]. Counselling the patients on lifelong treatment is crucial. Although a timed and careful withdrawal of drugs in other epileptic seizures leads to successful remission of the disease, in JME there is a 90% chance of recurrence with discontinuation of the drugs [5]. The first-degree relative of JME has an estimated 6% risk of developing epilepsy or suffering from various epileptic syndromes. Another study stated that the risk of epilepsy to first degree-relatives is almost doubled when JME was associated with an absence of seizures [4]. Therefore, genetic counselling is necessary for these patients.

Psychological evaluation of the patient revealed stressors, as the patient was a victim of physical abuse in her school. The condition was duly reported to the parents and local authorities for appropriate actions. As the MMSE of the patient was 23 with recent memory loss, it can be safely assumed that JME can present with cognitive dysfunction, which can either be due to an organic disease of the brain or simply due to psychological roots.

JME patients with poor seizure control, show impulsive decision making, and poor learning skills [6]; therefore, it is important to keep these patients in a seizure-free state. Long-term complications can be prevented with proper counselling, medical management, and avoidance of trigger factors. It has been reported that up to 75% of patients with JME can also suffer from psychiatric disorders, particularly anxiety, mood disorders, and cluster B personality disorders later in life [6]. Thus, a complete workup of JME patients should always include both psychiatric and psychological evaluation to administer appropriate management.

## Conclusions

Juvenile myoclonic epilepsy (JME) is a complex disorder which can sometimes be presented with atypical symptoms of cognitive dysfunctions. Extensive knowledge of history, clinical presentation of JME, and good command of the interpretations of diagnostic tools, along with comprehensive neuropsychological assessment, can help reduce misdiagnosis. JME patients are at major risk of developing psychiatric complications later in life; therefore, regular follow-ups and routine psychological assessment are beneficial in preventing such complications.

## References

[1] Katchanov J, Birbeck GL (2012). Epilepsy care guidelines for low- and middle-income countries: from WHO Mental Health GAP to national programs. BMC Med.

[2] Camfield CS, Striano P, Camfield PR (2013). Epidemiology of juvenile myoclonic epilepsy. Epilepsy Behav.

[3] Shah S, Sher K, Sattar RA (2014). Clinical and EEG characteristics of juvenile myoclonic epilepsy. Pak J Med Sci.

[4] Santos BPD, Marinho CRM, Marques TEBS (2017). Genetic susceptibility in juvenile myoclonic epilepsy: systematic review of genetic association studies. PLoS One.

[5] Afra P, Adamolekun B (2012). Lacosamide treatment of juvenile myoclonic epilepsy. Seizure.

[6] Koepp MJ, Thomas RH, Wandschneider B, Berkovic SF, Schmidt D (2014). Concepts and controversies of juvenile myoclonic epilepsy: still an enigmatic epilepsy. Expert Rev Neurother.

[7] Janz D (1985). Epilepsy with impulsive petit mal (juvenile myoclonic epilepsy). Acta Neurol Scand.

[8] Panayiotopoulos CP, Tahan R, Obeid T (1991). Juvenile myoclonic epilepsy: factors of error involved in the diagnosis and treatment. Epilepsia.

[9] Satishchandra P, Sinha S (2010). Progressive myoclonic epilepsy. Neurol India.

[10] Park KI, Lee SK, Chu K, Lee JJ, Kim DW, Nam H (2009). The value of video-EEG monitoring to diagnose juvenile myoclonic epilepsy. Seizure.

